# Experience in the Psychotherapeutic Treatment of Eating Disorders in Children and Adolescents: A Brief Approach and EMDR Outcomes

**DOI:** 10.1192/j.eurpsy.2024.954

**Published:** 2024-08-27

**Authors:** L. López Gómez-Miguel, L. Herranz Núñez, L. Santolaya López, F. Benavides Rivero, A. Privado Aranda, M. González San José

**Affiliations:** Unidad de Salud Mental, Hospital Universitario de Toledo, Toledo, Spain

## Abstract

**Introduction:**

This study is based on our experience at public hospitals and private clinics of Toledo and Madrid, where we have addressed the treatment of children and adolescents presenting with Eating Disorders (EDs). Our intervention focuses on the application of brief psychotherapy, with particular emphasis on the effectiveness of Eye Movement Desesitization and Reprocessing (EMDR) in these cases.

**Objectives:**

The primary objective of this study is to determine the benefits of applying EMDR in cases of pediatric and adolescent EDs in comparison to other psychotherapeutic techniques.

**Methods:**

Over a period of one year, brief psychotherapy sessions were conducted with children and adolescents diagnosed with EDs. An integrative approach was used, combining family sistemic therapy, cognitive-behavioural therapy techniques, and brief psychodynamic approaches, along with EMDR sessions. Pre and post treatment assessments were conducted to measure changes in symptoms and patients’ quality life.

**Results:**

The results obtained reveal significant improvements in patient symptomatology, including a notable reduction in food-anxiety, dietary restriction and compensatory behaviours. Furthermore, improvements were observed in body image perception and patiends’ overall quality of life. Incidence of relapse cases was minimal.

**Conclusions:**

Our experience suggests that the application of a brief psychotherapy approach, combined with EMDR sessions, can be highly effective in treating children and adolescents with EDs. Early intervention and individualized adaptation of therapies are essential for achieving positive and lasting outcomes in this patient group. These findings underscore the importance of considering integrative approaches in the care of EDs in young population.

**Disclosure of Interest:**

None Declared

